# P-118. Patient Reported Outcomes (PRO) Analysis of the Phase 2 Multi-Center, Prospective, Randomized, Double-Blind Study Assessing Nitazoxanide for the Treatment of Norovirus in Hematopoietic Stem Cell and Solid Organ Transplant Recipients

**DOI:** 10.1093/ofid/ofae631.325

**Published:** 2025-01-29

**Authors:** Catherine-Audrey Boutin, Michelle A Callegari, Diana F Florescu, Minh-Hong Nguyen, Daniel Kaul, Robin K Avery, Pearlie P Chong, Cynthia E Fisher, Ajit Limaye, Lisa A Clough, Steven A Pergam, Michael D Green, Marian G Michaels, Lara A Danziger-Isakov, Michael P Angarone, Amna Daud, Michael Ison, Laurie Keefer

**Affiliations:** Division of Infectious Diseases, Department of Medicine, Centre Hospitalier de l'Université de Montréal (CHUM), Montréal, Quebec, Canada; Northwestern University, Chicago, Illinois; University of Nebraska Medical Center, Omaha, Nebraska; University of Pittsburgh, Pittsburg, Pennsylvania; University of Michigan, Ann Arbor, Michigan; Johns Hopkins, Baltimore, Maryland; University of Texas Southwestern, Dallas, TX; University of Washington, Seattle, Washington; university of washington, Seattle, Washington; The University of Kansas Medical Center, Kansas City, Kansas; Fred Hutchinson Cancer Center; University of Washington, Seattle, WA; University of Pittsburgh School of Medicine, Pittsburgh, PA; UPMC Children's Hospital of Pittsburgh, Pittsburgh, Pennsylvania; Cincinnati Children's Hospital, Cincinnati, Ohio; Northwestern University Feinberg School of Medicine, Chicago, IL; Northwestern University Feinberg School of Medicine, Chicago, IL; Respiratory Diseases Branch, DMID/NIAID/NIH, Derwood, MD; Mount Sinai, New York, New York

## Abstract

**Background:**

Norovirus (NoV) results in relapsing, remitting diarrhea in immunocompromised hosts (ICH). No prospective studies of existing therapies including nitazoxanide (NTZ) have included evaluation of patient reported outcomes (PRO) or reported impact on patient quality of life (QOL).

**Figure:**
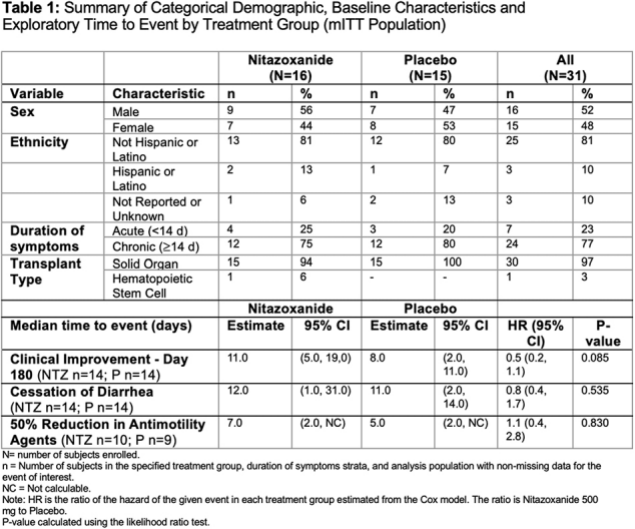

**Methods:**

We conducted a NIH-sponsored multi-center, prospective, randomized, double-blind study of NTZ for the treatment of NoV in adult HSCT and SOT recipients. Subjects with a positive NoV testing and active GI symptoms were randomly assigned (1:1) to NTZ 500 mg twice daily or placebo (P) for 56 consecutives doses and followed for 6 months. PRO diary assessments based on recognized questionnaires were collected: IBSQOL, EuroQOL, PROMIS Emotional, PROMIS GI, PANAS and PROMIS Physical Function Questionnaires.

**Figure 1.**
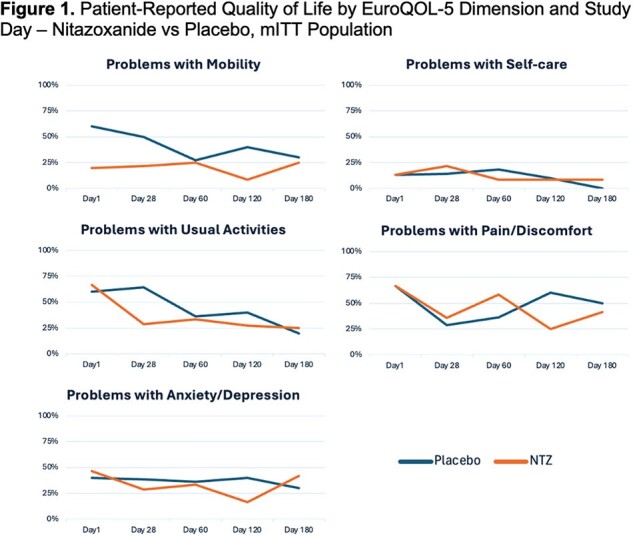
Patient-Reported Quality of Life by EuroQOL-5 Dimension and Study Day – Nitazoxanide vs Placebo, mITT Population

**Results:**

31 subjects (16 NTZ, 15 P) were enrolled (Table 1). Early withdrawal was documented in 5 subjects from each group. Thirty (30) had received solid organ transplants. Most had chronic (> = 14d) symptoms (77%). Based on data collected from PRO diaries, there were no significant between-treatment differences for change in diarrhea or nausea from baseline to day 180 (p=0.590 and 0.271). There was no significant difference in median time to 50% reduction in antimotility agents (NTZ 7d vs placebo 5d, p=0.830). However, the EuroQOL-5 questionnaire responses showed that patients seem to report less problems with anxiety/depression (NTZ 29% vs placebo 38%), mobility (21% vs 50%) and usual activities (29 vs 64%) at the end of study drug with NTZ (Fig. 1). There were significant difference in improvement of reported incontinence events between groups over time, which likely impact perceived QOL and activity level. Importantly, analysis of PROs demonstrates the significant and longstanding impact of NoV on ICH with high rates of anxiety/depression and reduced mobility at 180 days.

**Conclusion:**

PROs from this study provided significant insight into the expected course of NoV infection in ICH, including persistent but intermittent diarrhea, prolonged anxiety and depression, reduced mobility and reduced usual activity even out to 180 days post monitoring. This study provides data to support the use of PROs as primary endpoints in these studies.

**Disclosures:**

**Ajit Limaye, Professor/MD**, Memo: Advisor/Consultant|merck: Advisor/Consultant|merck: Grant/Research Support|moderna: Advisor/Consultant|moderna: site investigator|NobelPharma: DSMB member **Steven A. Pergam, MD, MPH**, Cidara: Advisor/Consultant|F2G: Advisor/Consultant|Global Life Technologies: Grant/Research Support|Symbio: Advisor/Consultant **Michael D. Green, MD, MPH**, ADMA: Advisor/Consultant|Bristol Myers Squibb: Advisor/Consultant|ITB-MED: Advisor/Consultant|kamada: Honoraria **Lara A. Danziger-Isakov, MD, MPH**, Aicuris: clinical research contract, paid to institutio|Ansun BioPharma: clinical research contract, paid to institution|Astellas: Advisor/Consultant|Astellas: clinical research contract, paid to institutio|Merck: clinical research contract, paid to institutio|Pfizer: Grant/Research Support|Takeda: clinical research contract, paid to institutio **Michael Ison, MD MS**, GlaxoSmithKline: Grant/Research Support|UpToDate: Royalties

